# Multi-slice CT coronary angiography assessment of remodeling index in patients with low- to intermediate-risk stable angina

**DOI:** 10.1186/s43044-019-0011-5

**Published:** 2019-09-11

**Authors:** Shaimaa A. Mostafa, Tarek Aboelazem, Osama Sanad, Haytham Abdelghafar, Ahmed Azam

**Affiliations:** 1 Cardiovascular Department, Faculty of Medicine, Benha Univerisity Hospital, Banha, Egypt; 2 Radiology Department, El-Agoza Police Hospital, Giza, Egypt; 3 Cardiovascular Department, El-Agoza Police Hospital, Giza, Egypt

**Keywords:** CT coronary angiography, Remodeling index, Stable angina

## Abstract

**Background:**

Early identification of vulnerable plaques by remodeling index prior to rupture and development of acute event is of considerable importance especially by a reliable non-invasive method as CT coronary angiography (CTA), so we aim to evaluate coronary artery remodeling index in patients with low- to intermediate-risk stable angina by CTA.

**Results:**

This single-center, cross-sectional, observational study included 150 patients with stable angina with normal resting ECG, negative markers, normal systolic function by 2D echocardiography (EF > 50%), and without regional wall motion abnormality at rest who were referred to MSCT evaluation of the coronary artery tree; the mean age was 56.8 ± 6.4 years, 83.3% had one-vessel disease, and 16.7% had two-vessel diseases. The mean remodeling index (RI) was 1.04 ± 0.28, 38% had significant positive remodeling, LAD was the most affected vessel (55.3), and proximal lesions were predominant in 48.5%; there was a statistically significant positive correlation between RI and cholesterol, triglyceride, LDL, duration of DM, HBA1c, and plaque burden (*P* < 0.001) and a statistically significant negative correlation with HDL (*P* < 0.001). Predictors of higher RI were positive family history, diabetes mellitus, low HDL, HBA1c, and plaque burden% (*P* < 0.001). Patients with remodeling index > 1.1 were diabetic, hypertensive, smoker, with longer duration of diabetes mellitus, higher HBA1c, cholesterol, triglyceride, LDL, plaque burden, wall lumen ratio, stenosis area, and lower HDL.

**Conclusion:**

CTA was able to detect the presence and extent of early, non-obstructive but significant coronary artery-positive remodeling in patients with low- to intermediate-risk stable angina patients.

**Trial registration:**

NCT03963609, 22 May 2019

## Background

The response of coronary arteries to atherosclerosis and plaque growth manifested as either compensatory enlargement or shrinkage which is known as coronary artery remodeling. Increase in vessel size due to the outward expansion of the vessel wall is called positive remodeling, while a decrease in vessel size due to vessel shrinkage is defined as negative remodeling [1].

The assessment of positive remodeling of coronary arteries has attracted a lot of attention due to the association of positively remodeled coronary plaques with plaque vulnerability and the propensity to cause future cardiac events based on histopathological and clinical studies [2].

Remodeling index is defined as the ratio of the maximum vessel area (or diameter) to a normal reference vessel area (or diameter), and plaques are classified as having significant positive remodeling when the RI is > 1.1 [3].

Although invasive coronary angiography is considered the gold standard for the diagnosis of coronary artery disease since it provides an excellent visualization of coronary lumen change, coronary angiography can only provide a two-dimensional outline of the coronary lumen and cannot demonstrate the complex nature of atherosclerotic plaques which are responsible for the association between the angiographic findings and clinical outcome [4].

Although IVUS is the standard reference for the assessment of coronary plaque composition and progression in clinical studies, it is an invasive procedure which is not commonly performed in routine clinical practice [4].

CTA has been widely used as an effective less invasive imaging modality to diagnose CAD as it allows the evaluation of coronary plaque characteristics and classification of coronary plaque compositions which has important clinical implications and association with myocardial ischemia [5].

It has been reported that almost two thirds of acute coronary events occur in noncritical lesions with less than 50% lumen stenosis, highlighting the necessity to detect and analyze these plaques at early stages, especially in low-risk patients, which would improve risk stratification without the need for more invasive procedures

CT imaging will be most useful in patients with an intermediate likelihood of CAS. In patients with low pretest likelihood, the false-positive rate may be too high, and in patients with high pretest likelihood, sensitivity may not be sufficiently high. Meijboom et al. have recently presented a careful analysis of the diagnostic value of coronary CTA, stratified according to the pretest likelihood of disease. They also found that the technique is most useful in patients with a low to intermediate likelihood of CAS [6].

## Aim

The aim was to evaluate coronary artery remodeling index in patients with low- to intermediate-risk stable angina by multi-slice computed tomography (MSCT) coronary angiography.

## Patients and methods

### Study design

The study design is a single-center, cross-sectional, observational study conducted in the period from January 2016 to January 2018; the study was approved by the local ethical committee and all patients signed informed consent.

All patients were subjected to thorough history taking, complete general and local examination, laboratory investigation, resting ECG, echocardiography, and then MSCT coronary angiography.

### Inclusion and exclusion criteria

#### Inclusion criteria

The study included adult patients, > 18 years of both genders with first attack, low- to intermediate-risk stable angina with normal resting ECG, negative markers, and normal systolic function by 2D echocardiography (EF > 50%) and without regional wall motion abnormality at rest who were referred to MSCT evaluation of the coronary artery tree.

#### Exclusion criteria

We excluded patients with a previous acute coronary syndrome or revascularization, those with renal impairment or dye hypersensitivity, patients with morbid obesity (BMI > 40 kg/m^2^), rhythm other than sinus rhythm, and inability to hold breath for 10 s to acquire the image and lesions with heavy calcium score.

### Echocardiography

All patients were examined in the left lateral position using (PHILIPS, EPIC 7C) machine with multi-frequency transducer. Standard views for two dimensional, M mode, Doppler, and tissue Doppler studies were obtained according to the recommendation of the American Society of Echocardiography for the assessment of left ventricular end-systolic and diastolic function.

### MSCT coronary angiography

The study was carried out on Aquilion Prime 320 slices Toshiba Medical System Corporation—Nishinasuno, Tokyo, Japan. The reassurance of the patients from the entrance to the scanning room was performed, including an appropriate knowledge of the whole process. A stable venous line should be available, this requiring an 18- to 20-gauge needle placed into an antecubital vein. The preparation for the study must include a pre-exam testing of the ability of the patient for sustaining a breath-hold long enough for the purposes of the examination. All patients received beta-blocking agent (ivabradine 5 mg once or twice if needed) to decrease heart rate to about 65–70 beats per minute.

Patients were placed within the gantry of the CT scanner in the supine position. According to the expected location of the coronary arteries obtained from the AP and lateral scout images, a preliminary scan without contrast injection was performed to determine the total calcium burden (calcium score) of the coronary tree; followed by ECG gated acquisition for coronary angiography with contrast material injected via a pump with total volume about 65–75 cc/patient. In all cases, the administration of the contrast was performed at a rate of 4–5 cc/s in order to more reliably enhance the vascular bed.

### Post-processing

The acquired images were transferred for post-processing on Vitrea WS—Vital Images, Minnetonka, Minnesota, USA. The most expansive atherosclerotic lesion in the original MDCT data sets was identified; serial multiplanar reconstructions (slice thickness 1 mm) were rendered in an orientation perpendicular to the longitudinal axis of the respective coronary artery segment. The cross-sectional vessel area was determined in a reference segment without detectable plaque proximal to and as close as possible to the respective coronary lesion (in absence of a segment without plaque, the least diseased segment between the lesion and the coronary ostium or major bifurcations), and several measurements were acquired including the lumen area, vessel wall surface area, and wall thickness, as well as wall percent area (wall thickness/vessel surface area) and wall/lumen percentage. Also, these measurements were repeated at the site of maximum arterial remodeling and compared with the reference segment measurements.

### Statistical methods

Data management and statistical analysis were done using SPSS vs. 25. Numerical data was summarized as means and standard deviations or median and ranges. Categorical data was summarized as numbers and percentages. Comparisons between patients with remodeling index (RI) less than or equal to 1.1 and patients with RI above 1.1 were done using independent *t* test or Mann-Whitney *U* test for normally and non-normally distributed numerical variables respectively. Categorical variables were compared using the chi-square test. Correlations were done using the Pearson or Spearman correlation if appropriate. “*r*” is the correlation coefficient. It ranges from − 1 to + 1; − 1 indicates a strong negative correlation, + 1 indicates a strong positive correlation, and 0 indicates no correlation. Linear regression analysis was done for the remodeling index as a dependent variable. All coefficients with 95% confidence intervals were calculated. All *P* values were two-sided. *P* values less than 0.05 were considered significant

## Results

During the period from January 2016 to January 2018, 225 patients with first attack stable angina were referred to MSCT coronary angiography, and only 150 patients were included for the assessment of the remodeling index.

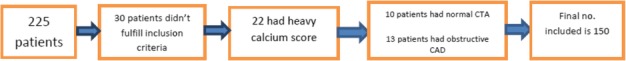



### Demographics and baseline criteria of the study population

The mean age of the study population was 56.8 ± 6.4 years; 87.3 % were male, 42.7% were hypertensive, 63.3% were smokers, and 55.3% were diabetic with a mean duration of diabetes (years) of 13.37 ± 4.49, and 43.3% had a family history of premature CAD. None of the patients was on statin therapy.

Mean serum cholesterol was 212.6 ± 33.8 mg/dl, mean TG was 213 ± 37.8 mg/dl, mean LDL was 97.5 ± 19.5 mg/dl, HDL was 42 ± 7.9 mg/dl, and mean HBA1c was 7 ± 2.6%.

By echocardiography, the mean EF% was 60.9 ± 5.5%. 27.3% of the study patients were referred before MSCT coronary angiography for a stress test (exercise ECG or stress echocardiography) that was not conclusive for stress-induced myocardial ischemia, but for the remaining patients, MSCT coronary angiography was the first choice.

### MSCT coronary angiography

#### The number of diseased vessels

83.3% had one-vessel disease and 16.7% had two-vessel disease; none of the patients had three-vessel disease or left main disease.

The LAD vessel was affected in 55.3% (44% as a single vessel and 11.3% as two-vessel disease), LCX was affected in 21.3%, and RCA was affected in 18.7% while the first diagonal and obtuse marginal were affected in 3.3% and 1.4% respectively.

Proximal lesions were predominant in 48.5%, then mid-segment lesions in 33.1%, and distal lesions in 6.8%.

#### Assessment of the affected segments

**Fig. 1:**
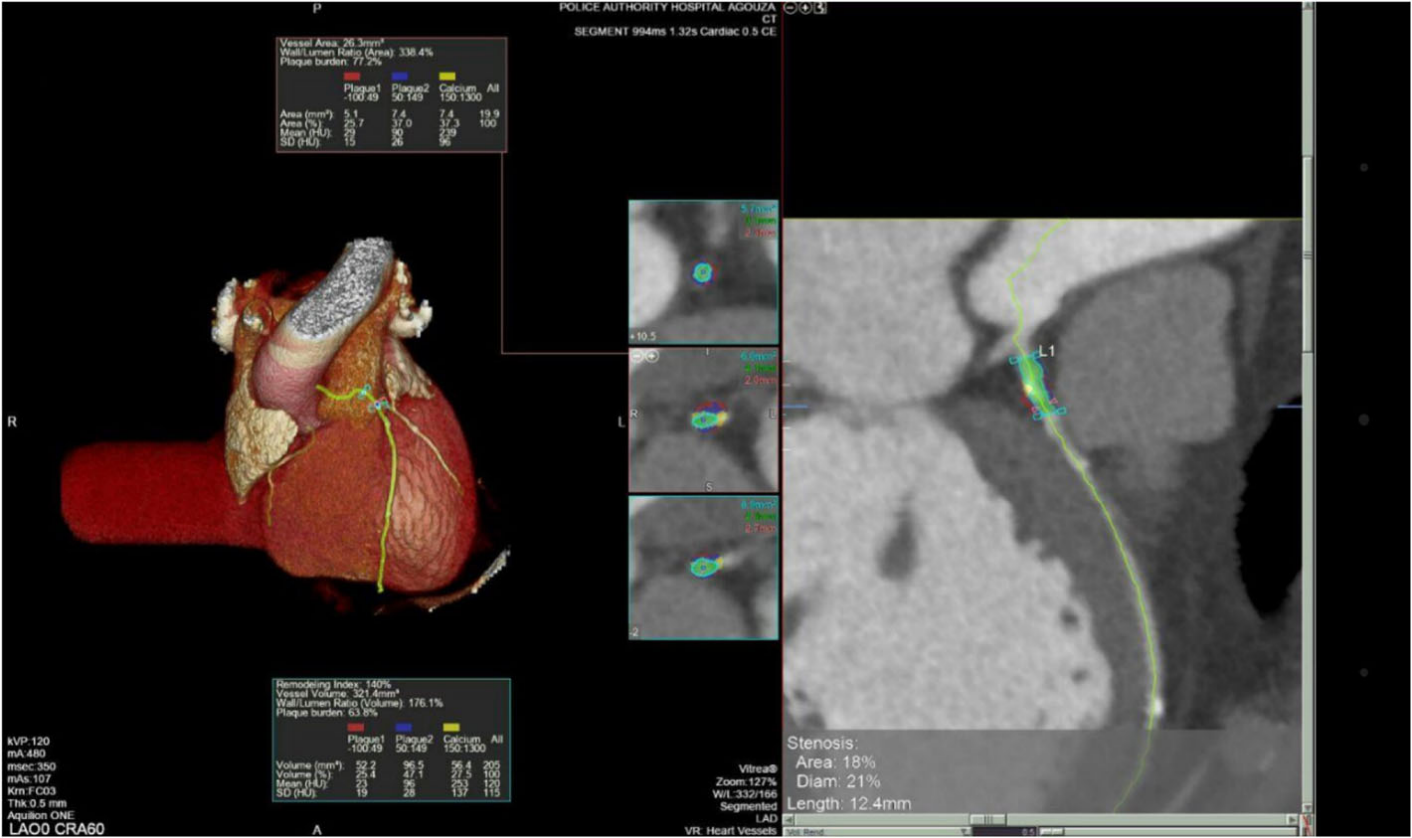
MSCT angiography coronary of a male patient, a 42-year-old smoker, and complaining of chest pain: HbA1c was 11, cholesterol level was 249, TGS 250, HDL 35, and LDL 100. CT angiography shows the proximal LAD lesion with remodeling index 1.4%, plaque burden 77.2%, wall lumen ratio 338.4%, stenosis area 18%, stenosis diameter 21%, and stenosis length 12.4 mm

The mean remodeling index was 1.04 ± 0.28, 38% had significant positive remodeling (RI > 1.1), mean plaque burden (%) was 53.3 ± 6.8, mean wall lumen ratio (%) was 147.1 ± 34.5, mean stenosis area (%) was 39.5 ± 16.7, stenosis diameter (%) was 24.2 ± 11.9, and length (mm) was 18.5 ± 10.6 (Fig. 1).

#### Correlation analysis for remodeling index

There was a statistically significant positive correlation between remodeling index and cholesterol, triglyceride, LDL, duration of DM, HBA1c, and plaque burden (*P* < 0.001) and a statistically significant negative correlation with HDL (*P* < 0.001) (Table 1).

**Table 1:** Correlation analysis for remodeling index (RI)

		RI
Age (years)	*r*	− 0.055
	*P* value	0.505
Duration of DM	*r*	0.518
	*P* value	< 0.001
HbA1c	*r*	0.663
	*P* value	< 0.001
HDL	*r*	−0.689
	*P* value	< 0.001
TGS	*r*	0.654
	*P* value	< 0.001
LDL	*r*	0.524
	*P* value	< 0.001
Cholesterol	*r*	0.652
	*P* value	< 0.001
Wall lumen ratio (%)	*r*	− 0.182
	*P* value	0.026
Stenosis area (%)	*r*	− 0.16
	*P* value	0.051
Stenosis diameter (%)	*r*	− 0.081
	*P* value	0.325
Length	*r*	− 0.08
	*P* value	0.33
Plaque burden (%)	*r*	0.531
	*P* value	< 0.001
LVED (%)	*r*	− 0.131
	*P* value	0.11

#### Predictors for high remodeling index

Multivariate linear regression analysis was done for prediction of remodeling index. Variance inflation factor (VIF) was used to assess collinearity. VIF of all factors were below 5, indicating no multicollinearity problems in the model.

By linear regression analysis; positive family history, diabetes mellitus, low HDL, HBA1c, and plaque burden% were predictors of high remodeling index (Table 2).

**Table 2:** Linear regression analysis for remodeling index (RI)

	*B*	SE	Beta	*t*	95.0% CI for *B*	*P* value
Constant	0.422	0.175		2.414	(0.076 to 0.767)	0.017
Age	− 0.001	0.002	− 0.022	− 0.577	(− 0.004 to 0.002)	0.565
Family history	0.122	0.023	0.221	5.26	(0.076 to 0.169)	< 0.001
HTN	0.022	0.022	0.039	0.98	(− 0.022 to 0.066)	0.329
DM	0.145	0.035	0.263	4.165	(0.076 to 0.214)	< 0.001
Cholesterol	0	0.001	0.049	0.629	(− 0.001 to 0.002)	0.53
Plaque burden (%)	0.011	0.002	0.278	6.655	(0.008 to 0.015)	< 0.001
TGS	0.001	0.001	0.141	1.901	(0.0004 to 0.002)	0.059
LDL	− 0.001	0.001	− 0.07	− 1.352	(− 0.002 to 0.0005)	0.179
HDL	− 0.01	0.002	− 0.274	− 5.872	(− 0.013 to -0.006)	< 0.001
HbA1c	0.015	0.006	0.14	2.343	(0.002 to 0.027)	0.021
Stenosis area (%)	0	0.001	− 0.011	− 0.279	(− 0.001 to 0.001)	0.781
Length	0.001	0.001	0.04	1.069	(− 0.001 to 0.003)	0.287

*B* regression coefficient, *Beta* standardized coefficient, *SE* standard error, *95% CI* 95% confidence interval

#### Comparison between study population with remodeling index ≤ 1.1 and > 1.1

Thirty-eight percent of the patients had remodeling index > 1.1, and when compared to those with RI < 1.1, they were found to have statistically higher prevalence of diabetes (100% vs. 28%, *P* < 0.001), hypertension (63.2% vs. 30.1%, *P* < 0.001), and smoking (86 % vs. 49.5%, *P* < 0.001) and longer duration of diabetes (14.41 ± 4.07 vs. 11.08 ± 4.59 *P* = 0.001).

Also, patients with remodeling index > 1.1 had statistically higher HBA1c, serum cholesterol, triglyceride, and LDL (*P* < 0.001) and lower HDL (*P* < 0.001).

By MSCT coronary angiography, there was higher plaque burden, wall lumen ratio, and stenotic area in patients with remodeling index > 1.1 (Table 3).

**Table 3:** Comparison between variables with RI ≤ 1.1 and > 1.1

		RI ≤ 1.1 (*n* = 93)		RI > 1.1 (*n* = 57)		
		Mean	± SD	Mean	± SD	*P* value
Risk factors	Age (years)	56.7	5.6	56.8	7.2	0.949
	Duration of DM (years)	11.08	4.59	14.42	4.07	0.002
	DM	No. (%)	26 (28)	No. (%)	57 (100)	< 0.001
	HTN	No. (%)	28 (30.0)	No. (%)	36(63.0)	0.001
	Smoking	No. (%)	46 (49.5)	No. (%)	49 (86.0)	< 0.001
Laboratory	HbA1c	5.8	2	9	2.3	< 0.001
	Cholesterol	196.9	28.4	238.2	25.3	< 0.001
	TGS	196.6	33.5	239.6	28	< 0.001
	LDL	90.5	15.5	108.9	20.1	< 0.001
	HDL	45.4	7.4	36.4	5	< 0.001
Echo	LVEF (%)	61.1	5.5	60.7	5.6	0.585
MSCT CA	Plaque burden (%)	51.2	6.2	56.6	6.3	< 0.001
	Wall lumen ratio (%)	153.9	35	136.1	31	0.002
	Stenosis area (%)	37.0 (7.0–76.0)		34 (3–76)		0.01
	Stenosis diameter (%)	23 (10–61)		21 (70.0–68.0)		0.348
	Length	15.5 (9.4–92.4)		14.9 (7.8–54.5)		0.683

## Discussion

Coronary artery disease is defined as clinically significant when luminal narrowing is present, typically at the 50% diameter reduction threshold. However, in early atherosclerosis, the first arterial changes consisted of compensatory enlargement of both the outer wall of the vessel as well as the lumen, termed compensatory enlargement or positive remodeling [7].

Positive remodeling had the best sensitivity and specificity (87% and 88%, respectively) as compared to low attenuation and spotty calcification to identify vulnerable plaque [8]. Being less conditional to image noise as plaque attenuation and having a more quantitative definition as the Napkin–ring sign, positive remodeling might become a more robust marker for vulnerable plaques [9].

Recent studies have demonstrated the incremental prognostic value of adverse plaque features over luminal stenosis. Comprehensive coronary plaque assessment holds the potential to significantly improve individual risk assessment [9].

The Promise trial found also that high-risk plaque was especially apparent among subjects with nonobstructive CAD on CTA (adjusted hazard ratio 4.31 vs. 2.64) among this low-intermediate-risk cohort (33% with 10-year atherosclerotic cardiovascular disease risk < 7.5%) [10].

So, the aim of the present study was the evaluation of coronary artery remodeling index in patients with low- to intermediate-risk stable angina by MSCT coronary angiography.

In the present study, 150 patients with coronary artery remolding fulfilled the criteria and were subjected to thorough history taking, laboratory investigation, ECG, resting echocardiography, and MSCT coronary angiography for the evaluation of the degree of remodeling index and its predictors and correlations.

83.3% had one-vessel disease and 16.7% had two-vessel diseases; none of the patients had three-vessel or left main disease. LAD was the most affected vessel followed by LCX and RCA, and the least affected were diagonal and obtuse marginal. Proximal lesions were predominant as well as mid and distal lesions.

The mean remodeling index was 1.04 ± 0.28. There was a statistically significant positive correlation between remodeling index and cholesterol, triglyceride, LDL, duration of DM, HBA1c, and plaque burden (*P* < 0.001) and a statistically significant negative correlation with HDL (*P* < 0.001). By linear regression analysis, positive family history, diabetes mellitus, low HDL, HBA1c, and plaque burden% were predictors of high remodeling index

Patients with remodeling index > 1.1 were diabetic, hypertensive, smoker, had longer duration of diabetes mellitus, higher HBA1c, cholesterol, triglyceride, LDL, plaque burden, wall lumen ratio, stenotic area, and had lower HDL (*P* < 0.001).

The finding of this study highlights two facts: the first is the careful consideration of symptoms even in patients with low to intermediate stable angina (normal ECG, good systolic function, no segmental wall motion abnormalities, negative markers of myocardial ischemia) and the second fact is the importance of correction of risk factors especially diabetes and dyslipidemia regarding the significant correlation with the remodeling index besides being predictors of positive remodeling.

The selection of the appropriate test to patients with low- to intermediate-risk stable angina is a matter of debate; in the present study, 27.3 % of the patients were referred before MSCT coronary angiography for stress test (exercise ECG or stress echocardiography) that was non-conclusive for stress-induced myocardial ischemia, but for the remaining patients, MSCT coronary angiography was the first choice.

In 2016, the National Institute for Health and Care Excellence (NICE), the organization that guides healthcare in the UK, updated its chest pain guideline and made coronary CTA as the first test for all patients without established CAD who present with typical or atypical angina or with non-anginal chest pain plus an abnormal resting electrocardiogram [11].

On the other hand, the current US stable ischemic heart disease guidelines favor noninvasive functional testing for myocardial ischemia in most patients, reserving anatomic testing using coronary computed tomography angiography (CTA) for patients without established CAD who have already undergone functional testing (inconclusive results or ongoing symptoms) or are unable to undergo functional testing [12].

The choice of noninvasive tests should always be individualized, accounting for local expertise, results of prior testing, and patient factors that influence test appropriateness and accuracy, but coronary CTA should at least always be an option available to patients and providers [13].

In the present study, proximal lesions were predominate which is an important finding regarding the sub-study of the PROSPECT trial which demonstrated that vulnerable plaques are frequently seen in the proximal coronary tree, followed by the mid-coronary tree and the least in the distal coronary tree [14].

The data of correlation of remodeling index with demographic and risk factors were variable; Burke et al. [15] found that positive remodeling was more in men and older group but Abdeldayem et al. [7] found that no correlation was noted between either age, sex, and coronary risk factors with remodeling index, but the latter result can be explained by the small number of the study population (35 patients) and so less prevalence of risk factors.

On the other hand, Britten et al. [16] found that cardiovascular risk factors like hypertension and hypercholesterolemia were associated with reduced positive or even negative remodeling. Moreover, the total number of classical cardiovascular risk factors was a strong predictor for reduced positive remodeling (*P* for trend < 0.001) meaning advanced stage of atherosclerosis from non-flow limiting to flow-limiting lesion as measured by fractional flow reserve.

We should consider that most acute coronary syndromes were initiated by sudden changes of mildly stenotic lesions, commonly found in positively remodeled arterial regions, rather than from progression of lesions already causing significant luminal narrowing; so, the identification of mildly stenotic but vulnerable atherosclerotic lesions and the overall plaque burden could provide better markers of coronary risk than measuring of luminal stenosis [7].

CT coronary angiography (CTA) has a sensitivity of 89% and specificity of 96% for the detection of presence, extent, angiographic severity, and composition of coronary atherosclerosis [17] even in early stage with positive remodeling which is difficult to be diagnosed by invasive coronary angiography which mainly examine the wall or by non-invasive test which unmask flow-limiting lesions. So, it enables to better identify patients most likely to benefit from aggressive preventive medications and lifestyle interventions.

## Conclusion

CTA was able to detect the presence and extent of early, non- obstructive, but significant coronary artery positive remodeling in patients with low- to intermediate-risk stable angina patients.

### Limitation

Besides being a single-center experience, a small number of patients also lack long-term follow-up to detect the outcome of patients with high positive remodeling or regression of the remodeling with control of risk factors.

## Data Availability

All materials are available.

## Abbreviations

CAD: Coronary artery disease
CTA: CT coronary angiography
MSCT: Multi-slice computed tomography
RI: Remodeling index
